# GNSS time-synchronised wireless vision sensor network for structural health monitoring

**DOI:** 10.1007/s13349-025-00953-7

**Published:** 2025-05-08

**Authors:** Miaomin Wang, Zuo Zhu, Ki Young Koo, James Brownjohn

**Affiliations:** Vibration Engineering Section, Faculty of Environment, Science and Economy, University of Exeter, Exeter, EX4 4QF UK

**Keywords:** Structural health monitoring, Computer vision, Wireless sensor network, Time synchronisation, GNSS

## Abstract

This paper presents the development of a time-synchronised wireless vision sensor network using the global navigation satellite system (GNSS). The sensor network, named the flexible vision network (FVN), offers significant advantages over existing wired or wireless time-synchronised vision sensor networks. These advantages include: 1) spatial flexibility, with no distance limitations between sensor nodes imposed by Ethernet cables or WiFi communication, 2) scalability in the number of nodes due to its independent time-sync operation based on satellites without any time-sync interaction with other nodes, and 3) straightforward time synchronisation with other heterogeneous sensor systems, such as accelerometers or dynamic strain systems, due to its independent time-sync operation providing an absolute time reference. A series of four laboratory experiments and one field experiment was conducted to validate the FVN, followed by an application experiment for simultaneous input–output measurements. The first laboratory experiment measured the timestamping error between two identical FVN nodes triggered by a common signal, finding a standard deviation of 17 µs in the timestamping difference. The second laboratory experiment assessed the timestamping error between two identical nodes tracking the same moving target, revealing a maximum time difference of 3.05 ms with rolling shutter cameras and 0.34 ms with global shutter cameras. This indicates that camera hardware significantly contributes to the error. The third laboratory experiment demonstrated a maximum displacement measurement error at 1/37 pixels level. The fourth laboratory experiment involved measuring time-synchronised displacements of 25 points on a laboratory floor structure using six nodes. The fifth field experiment measured displacements at 12 points along a footbridge. In both the laboratory and field experiments, the identified modal parameters were consistent with those obtained from wired acceleration measurement systems. The final experiment demonstrated a successful application of the FVN for time-synchronised input–output measurements in live pedestrian positioning and structural displacement, enabling the estimation of influence lines. While the experimental results were promising, the FVN requires clear visibility of the sky, which is generally achievable in field experiments involving civil infrastructure.

## Introduction

Vision-based systems offer a distinct advantage over contact-type sensors by capturing information of interest through an optical approach. This provides a contactless way to collect input loads and structural response data for structural health monitoring (SHM) [1]. Consequently, vision sensing techniques have gained popularity within the SHM community.

One notable application of vision-based systems is in measuring the displacement of structures such as bridges [2–5], buildings [6, 7], and dams [8]. These systems use cameras to record videos of structural vibrations, from which displacement data is extracted using various digital image processing algorithms, such as template matching [9–11], feature point tracking [12], optical flow [13], and motion magnification [14–16].

Another common application in SHM is in recognising and locating vehicles and pedestrians on bridges. For example, Pan et al. [17] proposed a vision-based framework to detect vehicles and estimate their tracklets, and this was successfully implemented in monitoring of the Jiangyin Bridge in China. However, merely collecting positional information of vehicles on bridges is insufficient for accurate load estimation. Various vehicle weighing methods have been suggested, including integrating vision-based vehicle detection with weigh-in-motion (WIM) [18] systems, and combining tyre–road contact models with computer vision techniques [19, 20].

When using a single-camera system, there is an inverse relationship between measurement accuracy and the camera's field of view (FOV), posing a challenge for accurate full-field measurement of large structures. Single-camera setups may struggle in multi-task and multi-scale scenarios, such as simultaneous measurements of input loads (like vehicles or pedestrians) and structural responses. To address these challenges, researchers have started employing multiple vision-based systems. Key aspects of using multiple systems include achieving time synchronisation, data transmission and processing.

One simple method involves post-processing, where camera nodes are triggered manually at different times, and the recorded videos are later imported to a computer for image processing. The timing of these videos is aligned using tools such as audio signals [21] and overlapping FOVs [22]. However, this method has low synchronisation accuracy as it heavily depends on the sampling rate of the camera. To improve synchronisation accuracy, researchers have used wireless methods to trigger camera nodes simultaneously, such as radio frequency [23] or stroboscopic light devices [24]. However, the sampling intervals are controlled by a crystal oscillator in the camera, a temperature-dependent component. This results in significant time uncertainty in long-term operations without corrections on the camera’s local clock by an external accurate time source [25].

Camera nodes can communicate with a master node/computer via cables. Specifically, the node/computer sends trigger signals to camera nodes simultaneously for recording video streams via a general-purpose input/output (GPIO) cable, and then the video data is transmitted to the master computer for image processing via an Ethernet cable. This setup provides a reliable method for time synchronisation and data transmission and processing, commonly adopted in commercial systems. However, the number of camera nodes is significantly limited by the data processing capability of the master computer. For instance, the Imetrum Dynamic Monitoring Station supports up to four camera nodes per master computer [26], and the distance between camera nodes and the master computer is constrained by cable length [27], nominally 100 m. These limitations challenge these systems in applications requiring large regional deployment of camera nodes.

To overcome the limitation of wired approaches, Shajihan et al. [28] have developed a wireless vision sensor network, SmartVision, for real-time multiple-point displacement monitoring of railway bridges. In this network, a wireless smart sensor platform (Xnode [29]) which uses a packet-based method is employed for time synchronisation. Specifically, a master node sends a packet including its precise current time information to the leaf nodes through radio waves, which use this information to update their local clocks. However, this process may become less efficient as the number of nodes increases, owing to network bandwidth limitations and overhead processing on the master node [30, 31]. The effective distribution range of camera nodes could be constrained by the intensity of the radio signals sent by the master node, resulting in spatial limitations on node deployment in complex SHM applications.

Another issue with existing wired or wireless vision sensor networks is the challenge of time synchronisation with other heterogeneous sensor systems, such as accelerometers or dynamic strain systems. This problem arises because different manufacturers often use various protocols for time synchronisation. As a result, synchronising multi-type sensor systems becomes difficult in SHM applications.

This paper presents a wireless time-synchronised vision sensor network, known as a flexible vision network (FVN). Each node within FVN independently synchronises its local clock with the atomic clock in global navigation satellite system (GNSS) satellites. This eliminates the need for a master node and inter-node communication, enabling FVN to operate without spatial deployment and node count limitations. The GNSS-based synchronisation method offers a simple way to synchronise with other heterogeneous sensor systems, as it relies on an independent time synchronisation process using satellites as an absolute time reference. Additionally, FVN consists of two types of nodes. Displacement nodes (DNodes) use a template matching algorithm for measuring displacement, while load nodes (LNodes) employ an object recognition algorithm to estimate the location of live loads. This facilitates multi-task SHM applications such as input–output measurement.

The novelty of this paper is to address the limitations of the existing wired and wireless vision sensor network, such as constraints on special deployment and node quantity, by developing the FVN. The developed vision sensor network features a unique node design that allows for time-synchronised input–output measurements of live load positioning and structural displacement responses. It provides a flexible, low-cost, and contactless solution for large-scale infrastructure SHM applications.

The rest of this paper is organised as follows: Sect. 2 introduces the hardware and software components of FVN. In Sect. 3, the synchronisation errors between FVN nodes are estimated, and the displacement measurement accuracy of a single node is verified. Section 4 presents a modal test conducted on a full-scale floor structure in a laboratory, while Sect. 5 showcases the efficacy of FVN in modal testing through an application on a real-life arch footbridge. Finally, Sect. 6 conducts a test to position live loads and the corresponding structural response using two FVN nodes, demonstrating their effectiveness in input–output measurement.

## Flexible vision network (FVN) for non-contact SHM

This section details the general setup of FVN for SHM applications first. The hardware components of an FVN node are described then, followed by an explanation of the time synchronisation process where each node aligns its local clock with a satellite clock via NTP. With the aspect of software, DNodes feature a subpixel resolution template matching algorithm for precise displacement measurement, while LNodes use an object recognition algorithm for vehicle and pedestrian positioning. The advantages of FVN are highlighted at the end of this section.

### General deployment setup

**Fig. 1:**
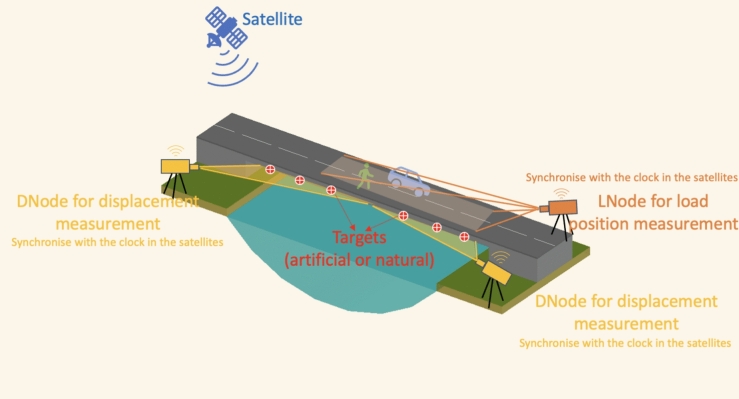
Overview of the FVN setup for SHM applications

Figure 1 illustrates a general FVN setup for SHM, with a focus on bridge monitoring. Vision-based systems face challenges in accurately measuring displacements under a large camera FOV. This limitation is especially significant when using a single-camera system to accurately capture structural displacements across an entire bridge. To enhance measurement accuracy, test points on the bridge are divided into sections, each monitored by an individual camera with a smaller FOV. Leveraging the FVN's flexibility in node distribution and heterogeneity in task processing, two DNodes are positioned on the opposite sides of the river to provide comprehensive coverage. Additionally, an LNode is dedicated to estimate the locations of vehicles and pedestrians, aiding in the estimation of live load effects on the bridge. Each node independently synchronises its local clock with the same atomic clocks in satellites, creating a time-coordinated sensor network. Captured images are locally processed, minimising the need for inter-node communication, and allowing the collection of vital structural data from multiple FVN nodes. This comprehensive data collection enables a thorough understanding of the structure's operational characteristics.

### Hardware of an FVN node

**Fig. 2:**
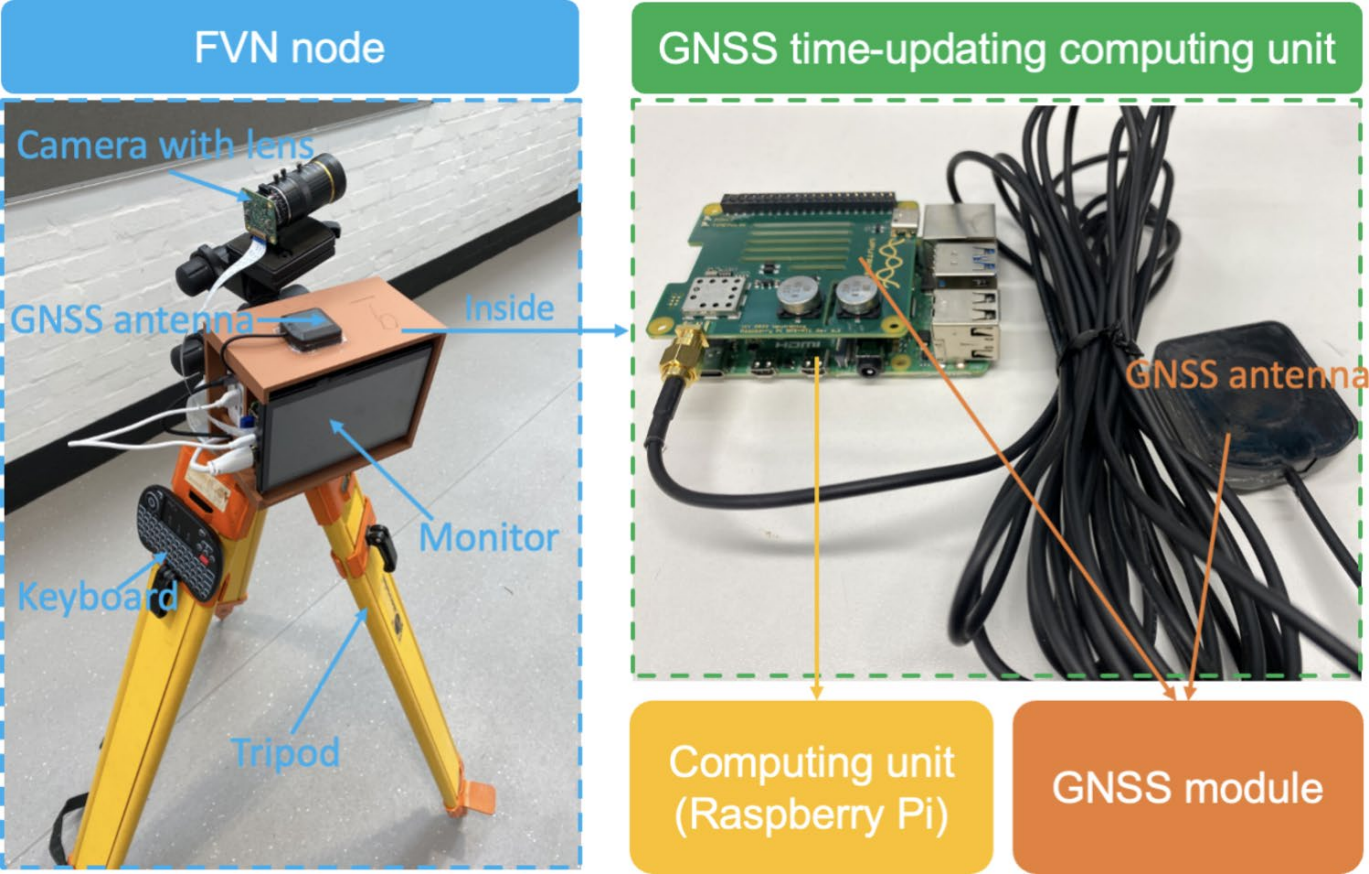
Hardware of an FVN node

In FVN, the hardware of each node (regardless of DNodes or LNodes) is generally the same, with different camera lenses used for different tasks. An FVN node, as illustrated in Fig. 2, consists of an image acquisition device, an image processing unit, a GNSS module, and various peripherals. Each FVN node is designed to be portable and cost-effective.

1. Image acquisition: The Raspberry Pi High Quality Camera, which has a high pixel resolution of 4056 * 3040 and is an affordable price, is used. A manual-zoom lens with a variable focal length of 8–50 mm is employed to meet specific requirements. For capturing high-speed objects, the Raspberry Pi Global Shutter Camera is preferred due to its ability to clearly image with global shutter sensors. The rolling shutter camera scans images line by line, causing distortions in fast motion, while the global shutter camera captures the entire image simultaneously, thus preventing such distortions.
2. Computing unit: The Raspberry Pi 4B, a single-board computer, is chosen for its balance between the computational capability and cost-effectiveness. This model features a Broadcom BCM2711 Quad-core Cortex-A72 64-bit SoC and 8GB of memory, which is suitable for real-time image processing.
3. GNSS module: The Uputronics Raspberry Pi GPS/RTC Expansion Board and the corresponding GNSS antenna provide high-precision timekeeping. Communication between the expansion board and the Raspberry Pi is facilitated through a Universal Asynchronous Receiver/Transmitter (UART) and pulse-per-second (PPS) pins.
4. Display and power: A portable 7-inch monitor is used for display purposes. The Raspberry Pi, GNSS module, and monitor are powered by a 50,000 mAh power bank, housed within a specially printed box, excluding the camera, to prevent motion caused by vibrations.
5. Type and control: Each FVN node is operated using a wireless keyboard and mouse.

### Software of FVN nodes

The software of each FVN node comprises two primary components: time synchronisation and image processing. Time synchronisation is accomplished through a combination of a GNSS module, NTP service daemon, and a user-modified driver. For image processing, a displacement calculation program using a template matching algorithm was developed for DNodes, while a load positioning program based on an object recognition algorithm was developed for LNodes. These programs, written in C++, operate on Raspberry Pi OS and utilise the OpenCV library for enhanced functionality.

To function correctly, each FVN node must be positioned so that its GNSS antenna has an unobstructed view of the sky. First, the Raspberry Pi's system clock is synchronised with satellite clocks using the GNSS module and NTP, creating a GNSS time-updating computing unit. Next, the node's camera begins capturing video streams. These frames are transmitted to the Raspberry Pi within the GNSS time-updating unit, where they are timestamped. In the final step, the timestamped frames are processed.

#### Raspberry Pi’s system clock synchronisation with satellite clocks via the GNSS module and NTP

GNSS is a satellite-based navigation system consisting of a network of satellites orbiting the Earth, each equipped with highly precise atomic clocks. These clocks are central to the system's accuracy, providing time information with nanosecond precision. Their exceptional accuracy makes them crucial for international time distribution services and various research fields. In this study, these atomic clocks are used for time synchronisation across all FVN nodes.

**Fig. 3:**
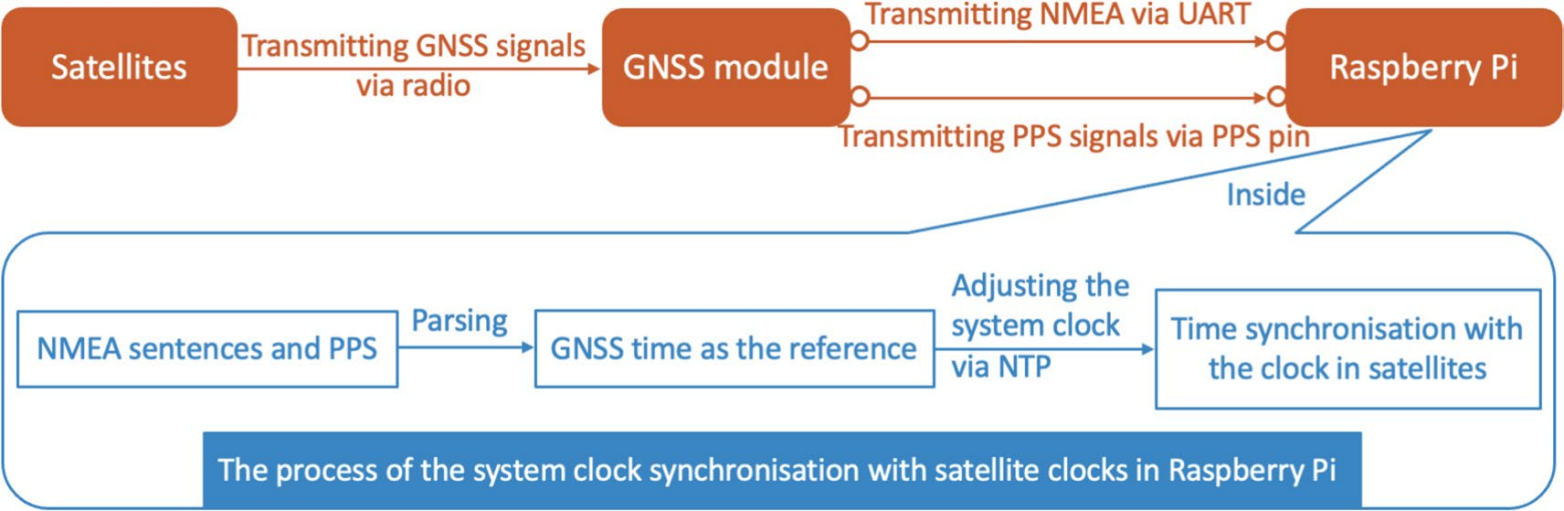
The process of synchronising the system clock of a Raspberry Pi with satellite clocks

Figure 3 illustrates the process of synchronising the system clock of a Raspberry Pi with satellite clocks. Initially, a GNSS module is physically connected to the Raspberry Pi, typically via several pins such as UART and PPS pins. This module receives signals from multiple GNSS satellites, each transmitting signal containing time information in National Marine Electronics Association (NMEA) sentences and PPS signals.

In the Raspberry Pi, an NTP daemon is installed to interpret the NMEA sentences and PPS signals from the GNSS module and extract the time information. This software component continuously compares the extracted time data with the system clock of the Raspberry Pi. If discrepancies are detected between the system clock and the GNSS-derived time, the NTP software adjusts the system clock accordingly, ensuring synchronisation with the GNSS time. This process establishes a GNSS time-updating computing unit.

#### Timestamping for each video frame in GNSS time-updating computing unit

**Fig. 4:**
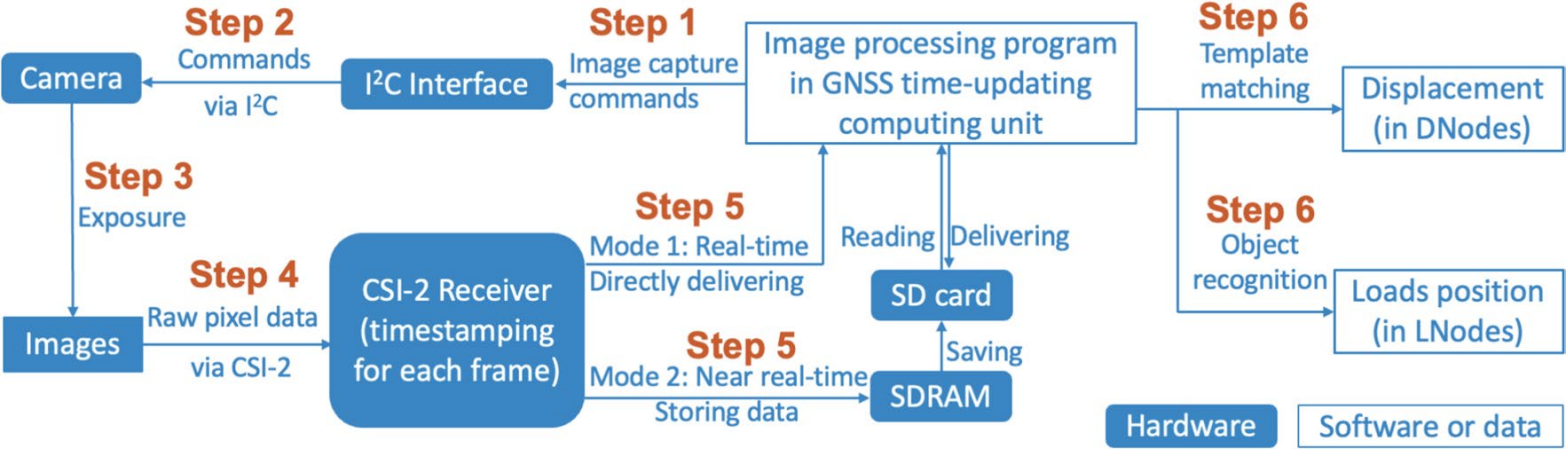
The process of image acquisition, transmission, and processing

After establishing a GNSS time-updating computing unit, the system initiates image capture and processing. Figure 4 illustrates the process of image acquisition, transmission, and processing. DNodes and LNodes follow the same process for image acquisition, transmission, and timestamping.

1. Step 1: The image processing program in the GNSS time-updating computing unit (Raspberry Pi) issues a capture command to the Inter-Integrated Circuit (I^2^C) interface.
2. Step 2: The command is transmitted to the camera sensor.
3. Step 3: Upon receiving this command, the camera starts an exposure to capture an image.
4. Step 4: The captured image is transmitted to the Camera Serial Interface 2 (CSI-2) receiver, known as Unicam. Here, each image is timestamped using the bcm2835-unicam driver.
5. Step 5: FVN supports both real-time and near real-time modes for data transmission. In real-time mode, timestamped image data is directly transferred to user space via the Video4Linux2 (V4L2) driver. In near real-time mode, the timestamped image is initially stored in a frame buffer, transferred to synchronous dynamic random access memory (SDRAM), and eventually written to an SD card in a video format. After recording, the image processing program retrieves and processes the video data from the SD card.
6. Step 6: The timestamped image is processed using a template matching algorithm for extracting structural displacement in DNodes and an object recognition algorithm for load positioning in LNodes.

In Step 4, the driver originally generated timestamps based on a monotonic clock, which was not updated by the NTP service, leading to unsynchronised data across the FVN nodes. To address this issue, the driver was modified and recompiled to use the system clock of the Raspberry Pi for timestamp generation. This ensures that each frame’s timestamp is based on a consistent time source, the satellite clocks.

In Step 5, each FVN node equipped with a rolling shutter camera supports both real-time and near real-time image processing modes. Nodes with a global shutter camera, however, only support near real-time mode due to the technical limitations of the camera. In scenarios like single-point displacement measurements, the real-time mode is typically utilised. This mode operates at a frame rate of less than 30 Hz, at a resolution of 1280*720, limited by the image processing speed of the computing unit. For applications requiring higher frame rates, the FVN node switches to near real-time mode, recording at a resolution of 1280*720 at 120 Hz with the rolling shutter camera or 60 Hz with the global shutter camera.

#### Structural displacement calculation in DNodes

DNodes are designed for structural displacement measurement. The camera should be positioned approximately perpendicular to the target on the structure. A scaling factor (SF), used to convert pixel displacement into physical displacement, is determined by the ratio of a known physical dimension to the pixel dimension in the captured image of the target.

**Fig. 5:**
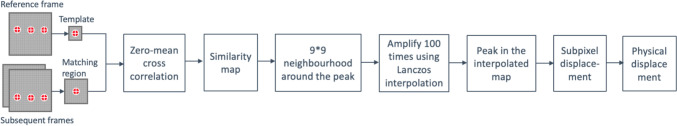
Workflow of structural displacement calculation in DNodes using the subpixel resolution template matching

When timestamped images are delivered to the user space, the subpixel resolution template matching algorithm is invoked to extract accurate displacement from the images. Figure 5 illustrates the workflow of the algorithm.

1. Definition of the template
  The first frame is considered to be the reference frame. A region of interest (ROI) containing the target is manually selected by the user as a template for tracking. The coordinates of the upper-left corner of the ROI are marked as (*x*^0^, *y*^0^). The width and height of the ROI are denoted by *w*_T_ and *h*_T_, respectively.

(2) Definition of the matching region
  To enhance the computational speed, a local region within subsequent frames is defined for template matching. This region, known as the matching region, has dimensions amplified by two factors, $\alpha$ and $\beta$ (both greater than 1), based on the template's dimensions. The width and height of the matching region are calculated as: ${w}_{M}={w}_{T}\times \alpha$ and ${h}_{M}={h}_{T}\times \beta$. The coordinates of this region in the current frame are updated from those in the previous frame.

(3) Calculation of zero-mean cross-correlation
  The zero-mean cross-correlation between the template and a corresponding region with the same dimension as the template (called the query window) within the matching region is calculated. The query window is an overlapped region determined by the template sliding across the matching region pixel by pixel. This correlation is calculated as:
  1 $$C=\frac{{(I}_{T}\left(x, y\right)-{I}_{{T}_{m}}){(I}_{Q}\left(x{\prime}, y{\prime}\right)-{I}_{{Q}_{m}})}{\Delta {I}_{T}\Delta {I}_{Q}},$$
  where ${I}_{T}\left(x, y\right)$ and ${I}_{Q}\left(x{\prime}, y{\prime}\right)$ are the greyscale intensities at the specific pixel within the template and query window, respectively; ${I}_{{T}_{m}}$ and ${I}_{{Q}_{m}}$ present the average value of the greyscale intensities within the template and query window; $\Delta {I}_{T}$ and $\Delta {I}_{Q}$ denote the standard deviations of the greyscale values within the template and query windows, respectively.

(4) Generation of the similarity map
  The zero-mean cross-correlation calculation is conducted for all positions of the query window within the matching region to form a similarity map. The dimensions of this map are $\left({w}_{M}-{w}_{T}+1\right)\times \left({h}_{M}-{h}_{T}+1\right)$. The coordinate of the peak in this map, marked as (*x*_*int*_^*i*^, *y*_*int*_^*i*^), indicate the target’s position in the *i*^th^ frame. However, the resolution of the coordinates is an integer pixel, which is not sufficient for accurate measurement applications. To enhance the accuracy beyond an integer-pixel resolution, an interpolation algorithm is used to resize the neighbourhood around the pixel.

(5) Definition of neighbourhood around the peak
  A 9 * 9 region around the peak of the similarity map is extracted for interpolation using the Lanczos algorithm, ensuring the peak is centred at the coordinates (4.5, 4.5).

(6) Amplification of the neighbourhood region
  The 9 * 9 neighbourhood region is resized to 900 * 900 using the Lanczos interpolation algorithm and amplified 100 times. The amplification factor can be adjusted by users based on specific applications.

(7) Determination of the peak in the interpolated neighbourhood region
  The coordinates of the peak in the interpolated region are denoted as (*X*_*sub*_^*i*^, *Y*_*sub*_^*i*^). The subpixel resolution coordinates of the peak are calculated as:
  2 $${x}^{i}={{x}_{int}}^{i}+\frac{{{x}_{sub}}^{i}}{100}-4.5,$$
  3 $${y}^{i}={{y}_{int}}^{i}+\frac{{{y}_{sub}}^{i}}{100}-4.5.$$

(8) Calculation of displacement in the frame
  The displacement between the target’s position in the current frame and the reference frame is determined as:
  4 $${{d}_{x}}^{i}={x}^{i}-{x}^{0},$$
  5 $${{d}_{y}}^{i}={y}^{i}-{y}^{0}.$$

(9) Determination of the physical displacement
  The displacement with subpixel accuracy in the frame is converted into physical displacement using the previously obtained *SF*. The physical displacements ${{(D}_{x}}^{i}, {{D}_{y}}^{i})$ are calculated as:
  6 $${{D}_{x}}^{i}={{d}_{x}}^{i}\times SF,$$
  7 $${{D}_{y}}^{i}={{d}_{y}}^{i}\times SF.$$

#### Vehicle and pedestrian positioning in LNodes

LNodes are used to estimate the locations of vehicles and pedestrians on structures. This task involves two main steps: (1) vehicles and pedestrians are first recognised and positioned within a timestamped image sequence using an object recognition algorithm; (2) subsequently, the pixel coordinates of the recognised objects are converted to their positions on the bridge deck.

This study employs the YOLO v8 (You Only Look Once, version 8) algorithm to recognise vehicles and pedestrians. YOLO v8 is a fast and accurate object detection algorithm that uses a single convolutional neural network (CNN) to simultaneously predict multiple bounding boxes and class probabilities for those boxes within an image sequence. For each bounding box, YOLO v8 provides the dimensions (height and width) and the centre coordinates, which are treated as the object’s position information within the image coordinate system.

To convert the position from pixel coordinates to actual positions on the bridge deck, the camera must be strategically placed to ensure the image plane aligns parallel to the bridge deck plane, as suggested in Ge’s study [32]. However, installing cameras in this orientation can be challenging in real-world bridge applications. A practical solution is to mount the camera on a tower overlooking the bridge deck, which is particularly feasible on cable-stayed and suspension bridges. In such a configuration, the position of objects on the bridge deck is converted to the image plane coordinates using a homography matrix ${\left[H\right]}_{3\times 3}$: $${\left[\begin{array}{ccc}{x}_{img}& {y}_{img}& 1\end{array}\right]}^{T}={\left[H\right]}_{3\times 3}{\left[\begin{array}{ccc}{x}_{phy}& {y}_{phy}& 1\end{array}\right]}^{T}$$, where ${x}_{img}$ and ${y}_{img}$ represent the object’s coordinate in the image plane, and ${x}_{phy}$ and ${y}_{phy}$ are the object’s position on the plane of the bridge deck. The homography matrix is resolved using at least four pairs of corresponding points with known locations in both the image plane and on the bridge deck. This method is adopted in this study for the scenarios mentioned above.

However, in bridge applications lacking tower-like structures, installing cameras with an overlooking view poses significant challenges. Typically, cameras are placed at the end of the bridge with a view approximately parallel to the cross section of the bridge girder, rendering the homography matrix method unsuitable for such a scenario. To address this limitation, this study simulates the concept of a depth camera to determine the three-dimensional (3D) coordinates of an object from its image coordinates, using a scaling factor method to estimate the depth.

First, the camera's intrinsic matrix and lens distortion parameters are established using Zhang’s calibration method [33]. The distance (i.e. the depth) $z$ between the object and the centre of the camera is estimated by using a similar triangle method: $z=D\times f/d$, where *D* is a known distance between two points in the 3D world coordinate system, *d* is the corresponding pixel distance on the pixel plane, and *f* is the lens focus length, which is already determined in the camera intrinsic matrix. Next, with the camera setup unchanged, the camera's extrinsic matrix is derived from at least four pairs of known locations in the pixel plane corresponding to the world coordinate system.

Once the necessary parameters are determined, the 2D coordinates of the object in the pixel plane are converted into 3D coordinates in the world coordinate system. First, the 2D pixel coordinates are transformed onto the normalised image plane by a reverse camera intrinsic matrix. They are then converted to 3D camera coordinate system by multiplying *z*. Finally, the 3D camera coordinates are transformed into 3D world coordinates using a translation vector and the reverse rotation matrix, which have been decomposed from the camera extrinsic matrix.

### Advantages of FVN

FVN offers several advantages for SHM applications:

1. Flexible time synchronisation: FVN nodes achieve precise time synchronisation, minimising spatial or node count limitations and allowing for more flexible deployment in complex SHM applications.
2. Diverse nodes: FVN consists of two types of nodes where DNodes measure structural displacement and LNodes identify and position moving loads on structures, facilitating comprehensive input–output SHM projects.
3. Straightforward synchronisation with other types of sensors: the GNSS-based method offers a simple way to synchronise with other types of sensor systems, as it relies on an independent time synchronisation process using satellites as an absolute time reference.
4. Non-contact measurement: FVN-based modal testing and input–output measurements can be conducted without needing access to operational bridges, avoiding disruptions to their normal use.
5. Independent operation: each FVN node independently completes image data acquisition and processing, enabling edge computing to directly output data and avoiding the need for large data transfers to a central master computer.

These advantages highlight the potential of FVN to enhance the efficiency and effectiveness of SHM in various infrastructure monitoring scenarios.

## Verifications on time synchronisation and measurement accuracy

This section describes three laboratory verification tests designed to evaluate the time synchronisation strategy and measurement accuracy outlined in this paper. Test 1 measured the synchronisation error between two GNSS time-updating computing units to verify the accuracy of the time synchronisation strategy used in this study. Test 2 estimated the time difference between displacement signals output by two FVN nodes (DNodes) measuring the movement of a shared target, demonstrating the time synchronisation performance of the FVN nodes. The results of Test 1 and Test 2 were then compared to quantify the effectiveness of the synchronisation strategy. Test 3 assessed the accuracy of displacement measurement of a DNode using a liquid crystal display (LCD)-based motion simulation technique.

### Test 1: synchronisation error between two GNSS time-updating computing units

The system clock of each Raspberry Pi in the computing units was synchronised with the satellite clocks via an NTP service. Consequently, the system clocks of all computing units were synchronised with each other. The accuracy of the synchronisation was vital for the reliable synchronisation of measurement data. To assess this, Test 1 evaluated the synchronisation error between the system clocks of two Raspberry Pis.

**Fig. 6:**
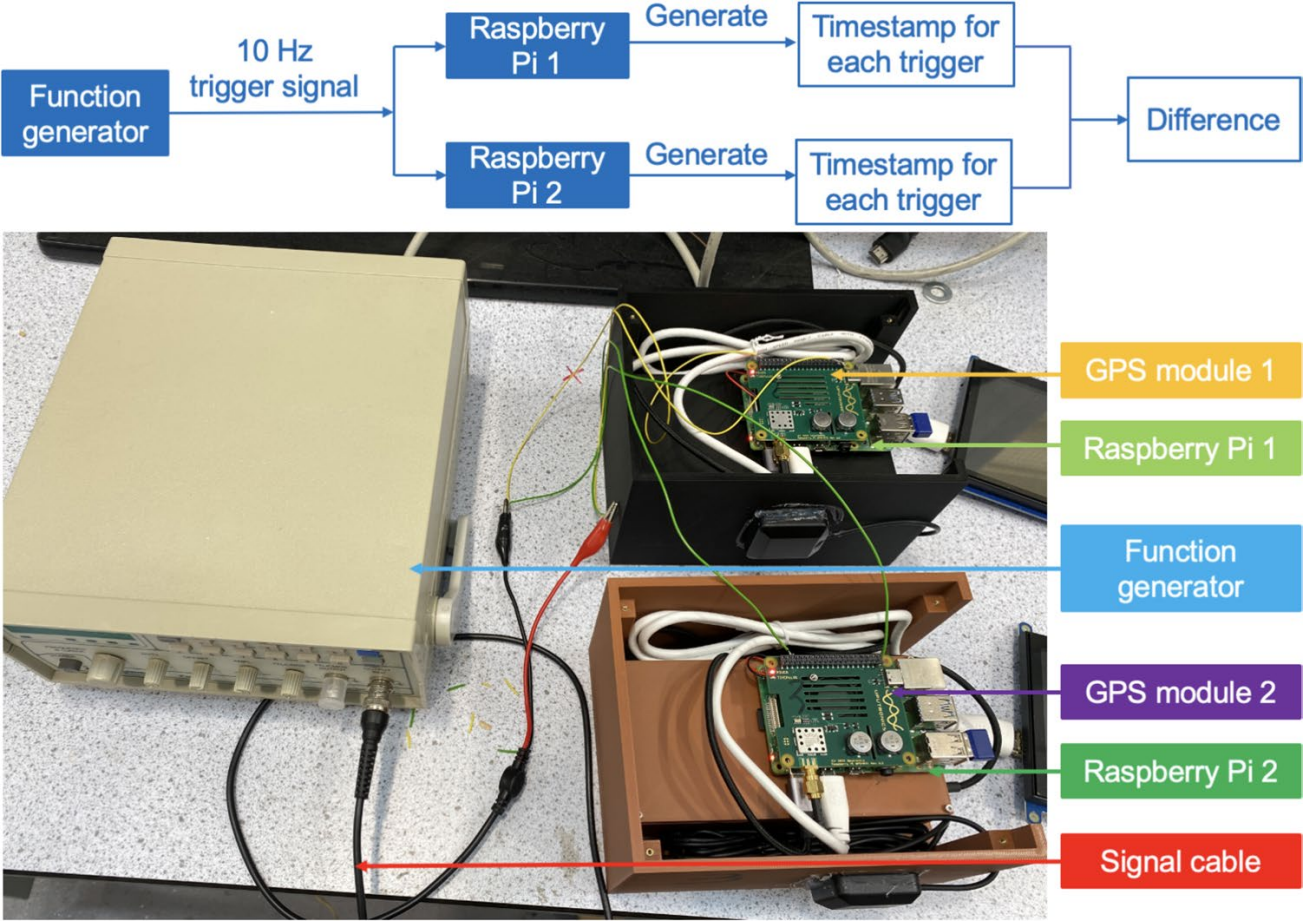
The setup of Test 1

Figure 6 illustrates the test setup. Two Raspberry Pis were connected to a function generator via cables. This function generator dispatched a 10 Hz trigger signal to the Raspberry Pis. Upon detecting the rising edge of each pulse, each Raspberry Pi generated a timestamp based on its system clock. It was assumed that the signal arrived simultaneously at both Raspberry Pis as the two signal paths had the same length. Any difference in the timestamps generated by the two Raspberry Pis would have indicated their synchronisation error.

Although FVN is mainly designed for field SHM applications, where the time source from the satellite clock is obtained via a GNSS antenna, indoor scenarios where GNSS signals are unavailable are also considered. This is addressed by accessing a public NTP time server through wireless Internet. The key difference between the two synchronisation methods is the time source: the first uses satellite clocks, while the second uses public time servers.

**Fig. 7:**
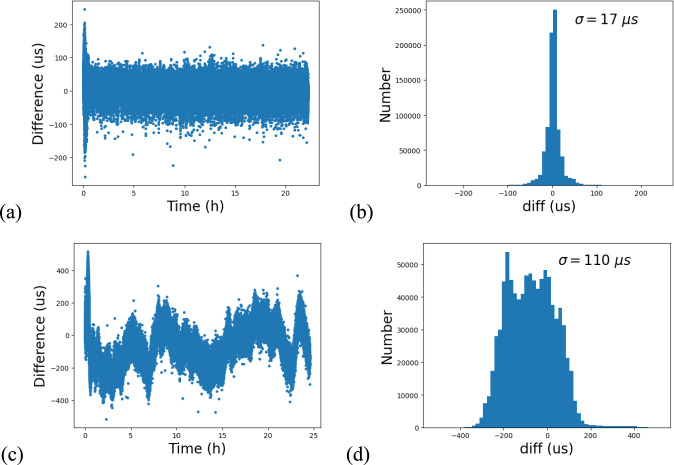
Synchronisation error between two Raspberry Pis in the GNSS time-updating computing units: **a** error distribution using the first method, **b** error histogram using the first method, **c** error distribution using the second method, and **d** error histogram using the second method

Synchronisation errors between two Raspberry Pis were measured over a 24-h period using both methods. The distribution and histogram plots of synchronisation errors for both methods are presented in Fig. 7.

Initially, as shown in Fig. 7a, larger synchronisation errors were observed, primarily because the NTP required some time to stabilise the system clocks. Once stabilised, the synchronisation error was predominantly controlled to under 100 microseconds (µs) when aligning with satellite clocks, achieving a standard deviation ($\sigma$) of 17 µs. In contrast, synchronisation errors fluctuated more when using the second method, yielding a significantly higher $\sigma$ of 110 µs, over six times that of the first method.

In Fig. 7b, it is evident that the synchronisation errors are symmetrically distributed around zero, indicating minimal systematic error when using the satellite clock method. Conversely, the histogram of synchronisation errors from the public time server method shows a noticeable bias in Fig. 7d.

The observed differences in synchronisation performance between the two methods can be attributed to their underlying mechanisms. The satellite clock method uses a direct time source through the NTP service, positioning each Raspberry Pi at the first stratum within this NTP hierarchy. However, the public time server method likely involves different public servers at various stratums, and the two Raspberry Pis may use public servers at different stratums. This could lead to a time offset of up to 100 ms between the public servers, resulting in a synchronisation bias. Additionally, the network paths to the respective NTP servers are different for each device, leading to fluctuating network latencies and larger time synchronisation errors.

### Test 2: time difference between displacement signals outputted by two DNodes

Camera sensors used for image acquisition involve stages of initialisation, exposure, and data transmission. These stages often require more time and introduce greater time uncertainties compared to analogue sensors, resulting in a time lag between when an image is captured and when the Raspberry Pi timestamps indicate the arrival of the image data. Consequently, there is a delay between the actual time of measurement and the timestamps produced by the Raspberry Pi. As a result, the time difference between data output by different FVN nodes is greater than the synchronisation error between two Raspberry Pis in the GNSS time-updating computing units.

DNodes and LNodes follow the same process for image capture, transmission, and timestamping. As a result, the time difference between data outputs from different FVN nodes is independent of the node types. In this test, two DNodes were used to estimate the time difference.

**Fig. 8:**
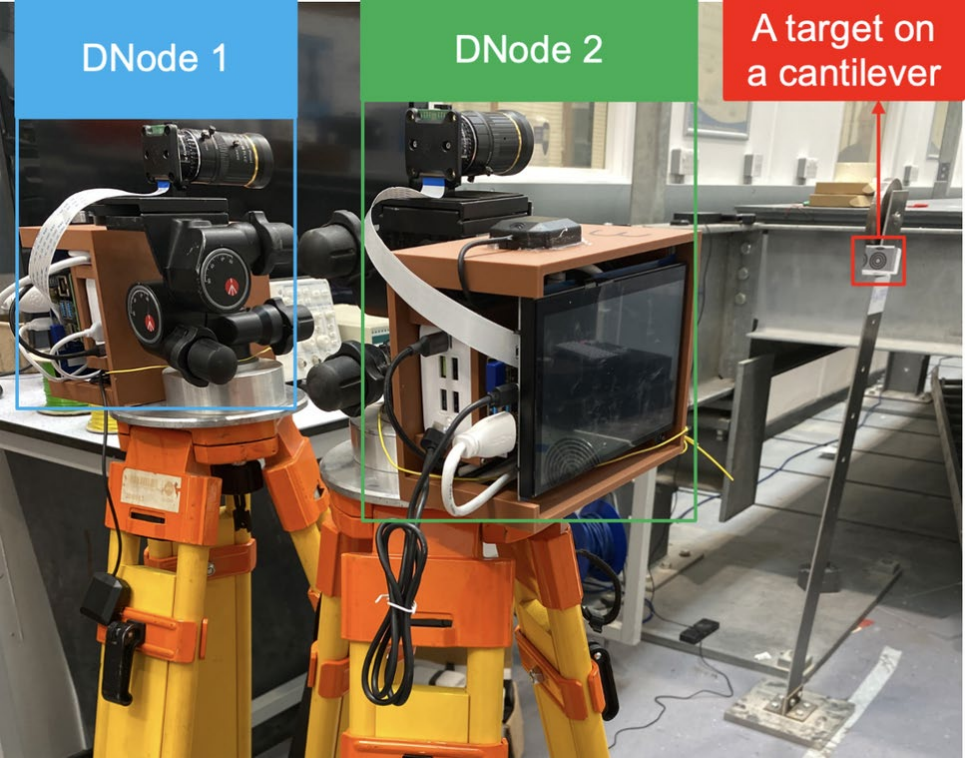
The setup of Test 2

This time difference can be influenced by various factors, such as the type of camera sensor and the time source for synchronisation. This study measures the time difference between displacement signals output by DNodes with different configurations to estimate their synchronisation performance in measurement data. Two types of cameras, rolling shutter and global shutter cameras, were used in this study, and their specifications are listed in Table 1. The test setup is presented in Fig. 8, and different DNode configurations are listed in Table 2.

**Table 1:** The specifications of the two cameras used in this study

Camera	Sensor	Shutter type	Used resolution	Pixel size ( µm)	Price (as of 2024)
Raspberry High Quality Camera	Sony IMX477	Rolling shutter	1280*720	1.55	£50
Raspberry Global Shutter Camera	Sony IMX296	Global shutter	1280*720	3.45	£50

**Table 2:** DNode configurations in Test 2

Case	Sensor	Frame rate (Hz)	Processing mode	Time source
1	Rolling shutter	20	Real time	Satellite clocks
2	Rolling shutter	100	Post-processing	Satellite clocks
3	Rolling shutter	20	Real time	Public time severs
4	Rolling shutter	100	Post-processing	Public time severs
5	Global shutter	50	Post-processing	Satellite clocks
6	Global shutter	50	Post-processing	Public time severs

Two DNodes were used to measure the dynamic displacement of a shared target on a cantilever in the laboratory. Despite being indoor, the DNodes could receive GNSS signals from satellites due to a partially glass roof on the laboratory building. Additionally, Wi-Fi access was available for the FVN nodes to synchronise with public time servers. The camera's frame rate was set to 20 Hz for real-time image processing mode in Cases 1 and 3 and increased to 100 Hz for video recording and subsequent post-processing in Cases 2 and 4. Cases 5 and 6 used the global shutter camera with a 50 Hz frame rate in the measurement.

The two DNodes began measuring the dynamic displacement of the target at different times. Subsequently, the structure was manually excited, inducing a period of free decay vibration. To accurately estimate the time difference between the obtained displacement signals, a coarse-to-fine method was employed. Initially, the time difference was estimated using the principle of maximum correlation between the two signals at the resolution of one sampling interval. Following this, the neighbouring values around the point of maximum correlation were interpolated using a second-order polynomial curve fitting. The peak of this polynomial curve was then calculated to determine the time difference with higher accuracy. The results of these time difference measurements under various conditions are presented in Table 3.

**Table 3:** Time difference between displacement signals output by DNodes in different cases

Case	Time difference in various test trials (unit: ms)					Mean
	Trial 1	Trial 2	Trial 3	Trial 4	Trial 5	
1	1.77	2.58	2.27	2.37	2.15	2.23
2	2.26	1.87	2.76	2.67	2.41	2.39
3	1.83	2.68	2.26	2.34	2.43	2.31
4	3.05	2.51	1.84	2.42	2.21	2.41
5	0.01	0.12	0.24	0.06	0.10	0.11
6	0.02	0.10	0.14	0.34	0.30	0.14

From the test results, it was evident that the type of camera sensor had the most significant influence on the time difference between displacement signals output by two DNodes. Specifically, the maximum time difference was 3.05 ms and the mean time differences exceeded 2 ms when employing the rolling shutter camera in the DNodes. Conversely, when utilising the global shutter camera, these differences were controlled to under 0.2 ms and the maximum time difference was 0.34 ms. This discrepancy can be explained as follows: In a rolling shutter setup, the sensor scans the image row by row, starting from the top and progressing to the bottom. Consequently, there is a slight delay between the capture of different segments of the image. In the case of the rolling shutter camera used in this study, the timestamp assigned to the image corresponds to the start of the scanning process, despite the target being in the central area of the image. This misalignment between the timestamp and the actual time of capturing the target arises due to the nature of the rolling shutter mechanism. Conversely, with global shutter technology, the entire sensor captures the image simultaneously, thus eliminating the delay associated with rolling shutter cameras and ensuring a closer alignment between the timestamp and the actual capture time.

As observed in Sect. 3.1, using satellite clocks directly as the time source yields a smaller synchronisation error between two Raspberry Pis compared to using public time servers. This observation is consistent with the findings presented in this section. Specifically, when transitioning from satellite clocks to public time servers as the time source, a slight increase in the time difference was observed, suggesting the existence of major timestamping uncertainty in the cameras used in the experiment.

### Test 3: measurement accuracy validation of a DNode

Multiple DNodes can collaborate to accurately measure the full-field structural displacement of large-scale infrastructures. To evaluate their performance, this subsection validates the measurement accuracy of a DNode.

**Fig. 9:**
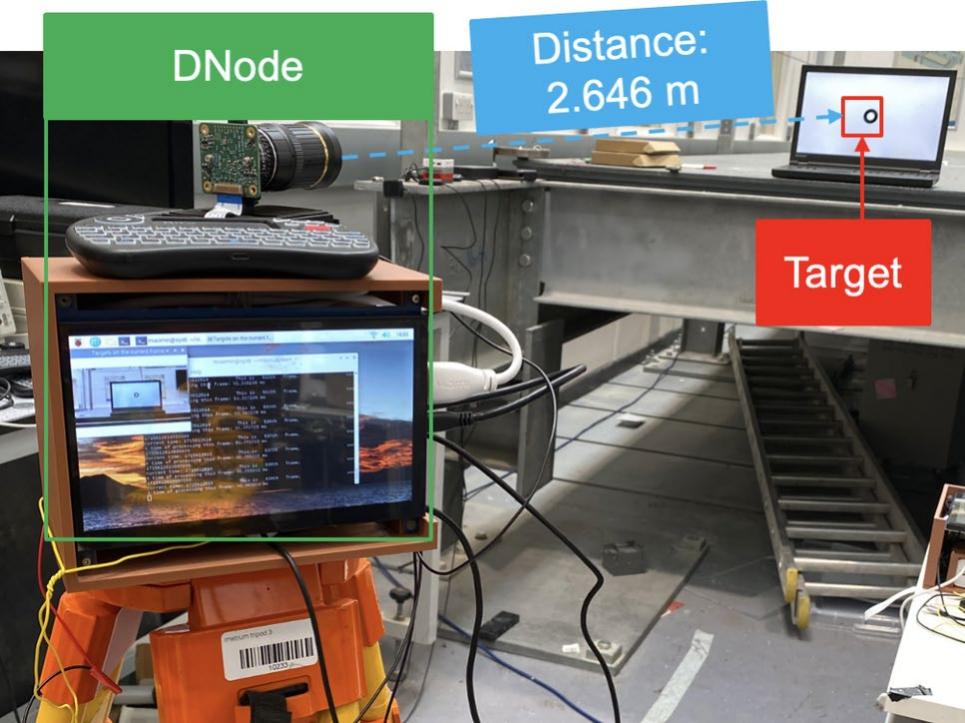
Experimental setup of Test 3

An experiment was conducted using a DNode to measure the displacement of a moving annulus pattern displayed on an LCD monitor, as illustrated in Fig. 9. A camera, mounted on a surveying tripod, was positioned 2.646 m from the monitor. The annulus pattern moved downwards in ten steps, pausing for 1 s at each step. Upon reaching the tenth step, it retraced its steps. The displacement between adjacent steps was five pixels on the monitor, equating to a physical displacement of 0.9 mm per step (with each pixel measuring 0.18 mm). This known displacement was used as a reference to estimate measurement errors by comparing the captured data against it.

**Fig. 10:**
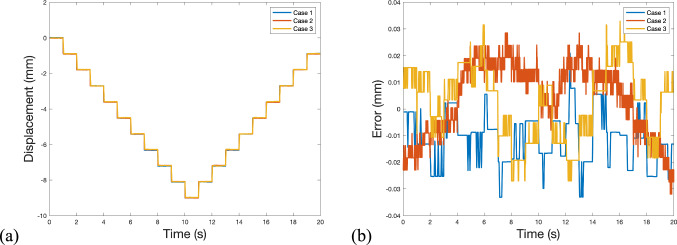
Measurement results of Test 3: **a** displacement time history and **b** measurement error

The evaluation involved three test scenarios with different camera configurations, such as Cases 1, 2, and 5 of Table 2. The three cases of this measurement accuracy test are detailed in Table 4. In Case 1, a rolling shutter camera operating at a 20 Hz frame rate was used in real-time measurement mode. In Case 2, the same camera recorded a video of 100 Hz frame rate for post-processing. Case 3 employed a global shutter camera to record a video of 50 Hz frame rate for post-processing. The FOVs in these configurations were adjusted by changing the camera zoom, resulting in scaling factors of 0.664 mm/pixel, 0.458 mm/pixel, and 0.783 mm/pixel, respectively. The results, including displacement time history and measurement errors, are shown in Fig. 10. The average measurement errors are listed in Table 4.

**Table 4:** Average measurement error of a DNode

Case	Sensor	Processing mode	Scaling factor	Average error (mm)	Average error (pixel)
**1**	Rolling shutter	Real time	0.664 mm/pixel	0.0126 mm	1/53 pixel
**2**	Rolling shutter	Post-processing	0.458 mm/pixel	0.0124 mm	1/37 pixel
**3**	Global shutter	Post-processing	0.783 mm/pixel	0.0120 mm	1/65 pixel

The DNode demonstrated precise displacement measurements across the three scenarios with varying camera configurations. In Case 1, the node achieved an accuracy of 1/53 pixels. In Case 2, the accuracy decreased to 1/37 pixels due to two main factors: the high sampling rate of 100 Hz, which resulted in insufficient exposure time and increased noise, and the degradation of image quality due to video encoding and compression.

Case 3 yielded the most accurate measurements, with an accuracy of 1/65 pixels, using a global shutter camera. This superior performance could be mainly attributed to the larger pixel size of the global shutter camera sensor (3.45 µm) compared to the rolling shutter camera (1.55 µm). Generally, a larger pixel size enhances the camera's imaging capability, leading to more accurate measurements.

## Modal testing of a full-scale floor structure in the laboratory

While vision-based modal testing has been successfully applied to many bridges [34, 35], conducting a modal survey on floor structures with small vibrations using a single vision system remains challenging. This difficulty arises from constraints in accurately measuring displacements within a large camera FOV and limited measurement regions due to the shallow depth of field. To address these challenges, this section presents an output-only modal test on a stiff floor structure using six DNodes. The results demonstrate the effectiveness of using multiple DNodes for dynamic tests on floor structures.

### Test setup

**Fig. 11:**
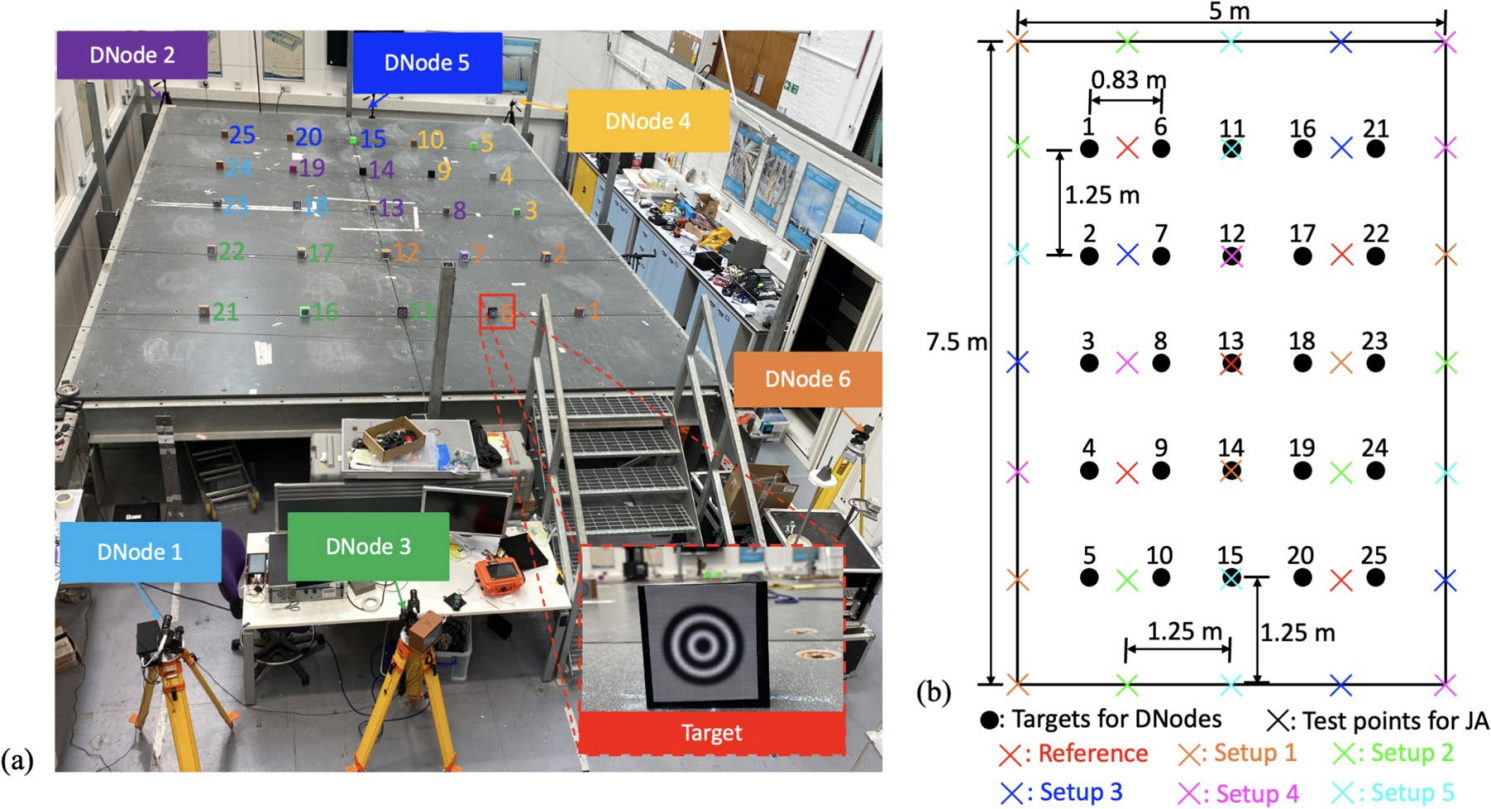
Test setup of the modal testing on a steel floor structure: **a** FVN nodes and corresponding targets, and **b** test points for JA accelerometers

This output-only modal test was conducted on a steel floor structure. The experimental setup is illustrated in Fig. 11. The structure measures 5 m by 7 m and is composed of twelve 1.25 m by 2.5 m steel plates, supported by 'I type' girders along all four sides and an additional 'I type' girder along the middle line where Targets 11-15 are distributed. Unlike a beam-like structure, the dynamic properties of this floor are more like those of plates. Some 25 test points were distributed across the structure, with an interval of 0.83 m along the width and 1.25 m along the length.

Six DNodes were used to cover all test points. Artificial targets were placed at the test points for vertical displacement measurement, as shown in Fig. 11a. The six DNodes were placed around the structure, each responsible for several specific targets, distinguished by different colours in Fig. 11a. For example, DNode 2 is marked in purple, corresponding to targets 8, 13, 14, and 19. The arrangement of these nodes was primarily determined by the available space around the structure. The nodes were synchronised using public NTP servers via the Internet. Given that the natural frequency of the floor is around 6 Hz, a high sampling rate was necessary to capture more structural modes. Each DNode camera was set to record a video at a resolution of 1280 by 720 pixels at 100 Hz. The displacement of the targets was then extracted from the recorded video.

The six nodes began recording videos at different times. During the test, the structure was excited by the participants performing heel drops, then remaining still until the vibrations decayed out. The length of the recording was approximately 20 min.

To evaluate the results based on the extracted displacement signals, an accelerometer system was also used. However, only 11 accelerometers were available for the test, necessitating an additional five setups of ambient modal testing following the vision-based testing. There were 35 test points on the structure for the accelerometer-based test, represented by cross symbols in Fig. 11b. The accelerometers used were Japan Aviation Electronics JA-70SA tri-axial MEMS accelerometers, and their signals were acquired by a Data Physics Abacus 906 data logger. Five accelerometers were used as references, while the other six sensors were used for the roving setups. The sampling rate of the accelerometers was 128 Hz and the length of the recording of each roving setup was 20 minutes. The layout of the sensors is shown in Fig. 11b.

### Test result

**Fig. 12:**
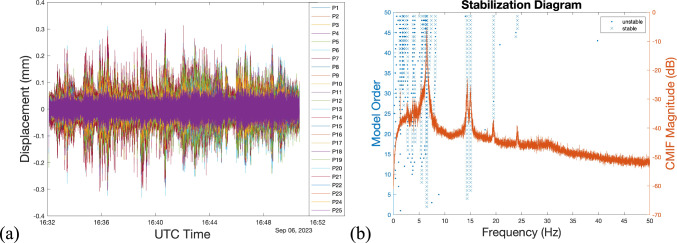
Test result **a** displacement time history of all test points, and **b** stabilisation diagram using the SSI-COV

The vertical displacement of all test points was extracted from the videos recorded by the DNodes. The results indicated minimal vibration in the floor structure, with a maximum amplitude of less than 0.4 mm. Since the six nodes started recording at different times, an overlapping duration among the extracted displacement signals was determined for analysis. To perform the modal identification algorithm on these signals, all data was resampled for alignment, and shown in Fig. 12a.

**Fig. 13:**
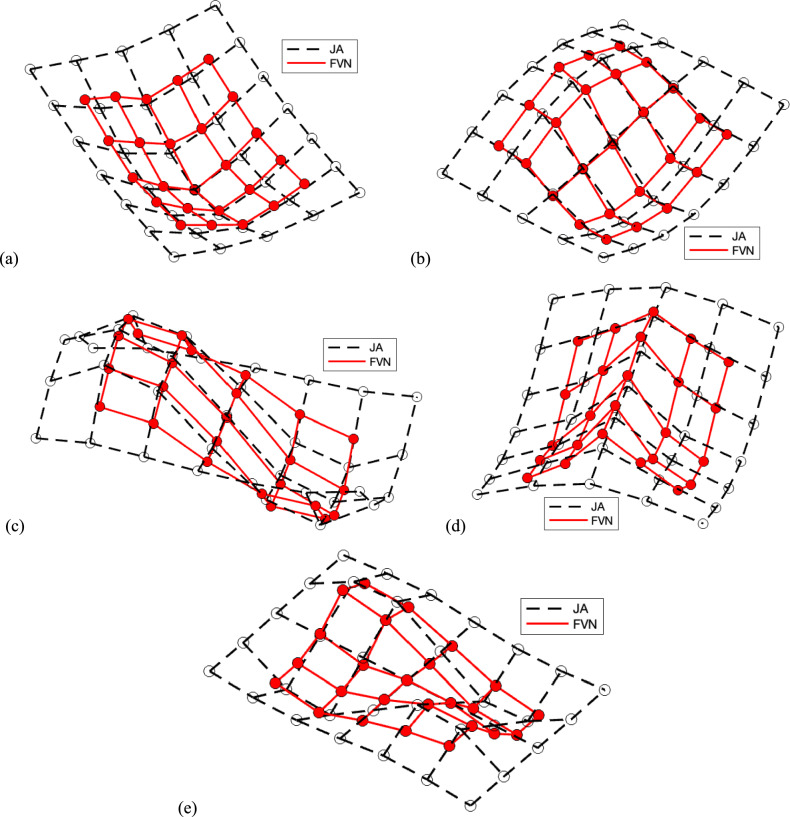
Mode shapes identified based on data obtained by FVN and JA: **a** Mode 1, **b** Mode 2, **c** Mode 3, **d** Mode 4, and **e** Mode 5

A covariance-driven stochastic subspace identification (SSI-COV) method [36] was then used to identify structural modes based on the resampled displacement data, obtaining a stabilisation diagram as shown in Fig. 12b. Five modes of the floor structure were identified, as shown in Fig. 13, with corresponding mode frequencies listed in Table 5. Mode 1 represents the first symmetric bending mode, with a natural frequency of 6.52 Hz. Modes 2 and 3 are the first asymmetric bending modes along the width and length, with frequencies of 14.38 and 15.04 Hz, respectively. Modes 4 and 5 have frequencies of 19.54 and 24.19 Hz.

**Table 5:** The structural frequencies identified based on data obtained by the FVN and JA systems

Mode	FVN	JA	Difference
1	6.52	6.52	0.00 %
2	14.38	14.41	0.21 %
3	15.04	15.13	0.59 %
4	19.54	19.08	−2.41 %
5	24.19	24.45	1.06 %

For validation, the SSI-COV method was also applied to the acceleration data, and the identified results served as a reference for evaluating the performance of the DNodes. The identified mode shapes from the acceleration data are plotted in Fig. 13, with corresponding mode frequencies listed in Table 5.

The differences between the identified mode frequencies from the two measurement systems are calculated and listed in Table 5. The first three differences are less than 1%, while the last two differences are −2.41% and 1.06%, respectively. These results from the lower three modes indicate that the identified mode shapes and frequencies from both FVN and accelerometer data matched well, demonstrating that FVN is capable of non-contact modal testing on plate or floor structures. However, the higher errors in the two highest modes are those expected, as the vibrational amplitudes of the higher modes are much smaller, resulting in higher error when measured by computer vision.

Similarly, the differences in modal amplitudes at higher frequencies were also larger. The DNodes use low-cost cameras, which have limited ability to accurately capture subtle vibrations compared to high-quality accelerometers. As a result, the signal-to-noise ratio (SNR) in the high-frequency displacement data was relatively low.

In this test, six DNodes were used to measure the displacement at all test points in a single setup, compared to the 11 accelerometers required. Additionally, five setups were necessary to capture the vibration of 35 test points with accelerometers. This indirectly demonstrates the advantages of using FVN in modal testing of plate or floor structures in terms of test time and cost compared to accelerometers.

## Modal testing of an operational arch footbridge

In general, bridges exhibit small vibrational displacements under operational loads. Capturing these small displacements requires a narrow camera FOV for higher measurement accuracy, limiting the range of points that can be measured by a single node. Consequently, using a single vision system to capture the vibrations of the entire structure is challenging. To address this issue, this section presents an output-only modal testing study on an operational arch footbridge using three DNodes, demonstrating the efficiency of FVN in modal testing of bridges.

### Test setup

**Fig. 14:**
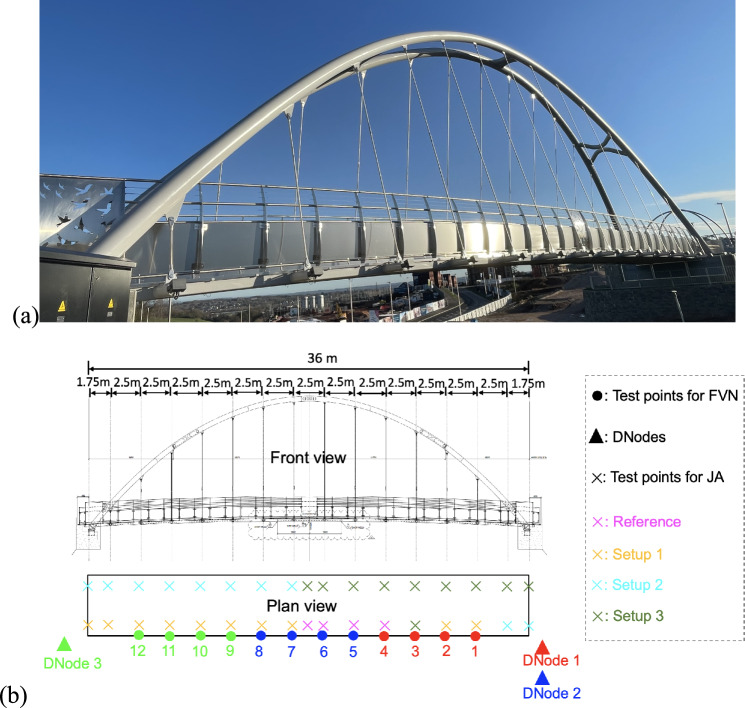
**a** Overview of the bridge and **b** layout of the test points and DNodes

The bridge tested is a steel through-tied arch bridge, designed for pedestrians and cyclists, located in Exeter, UK. It spans the A379 road between Chudleigh Road and the Devon Hotel, with a length of 36 m and a height of 10 m. The bridge deck is supported by 14 hangers on each side, as shown in Fig. 14.

**Fig. 15:**
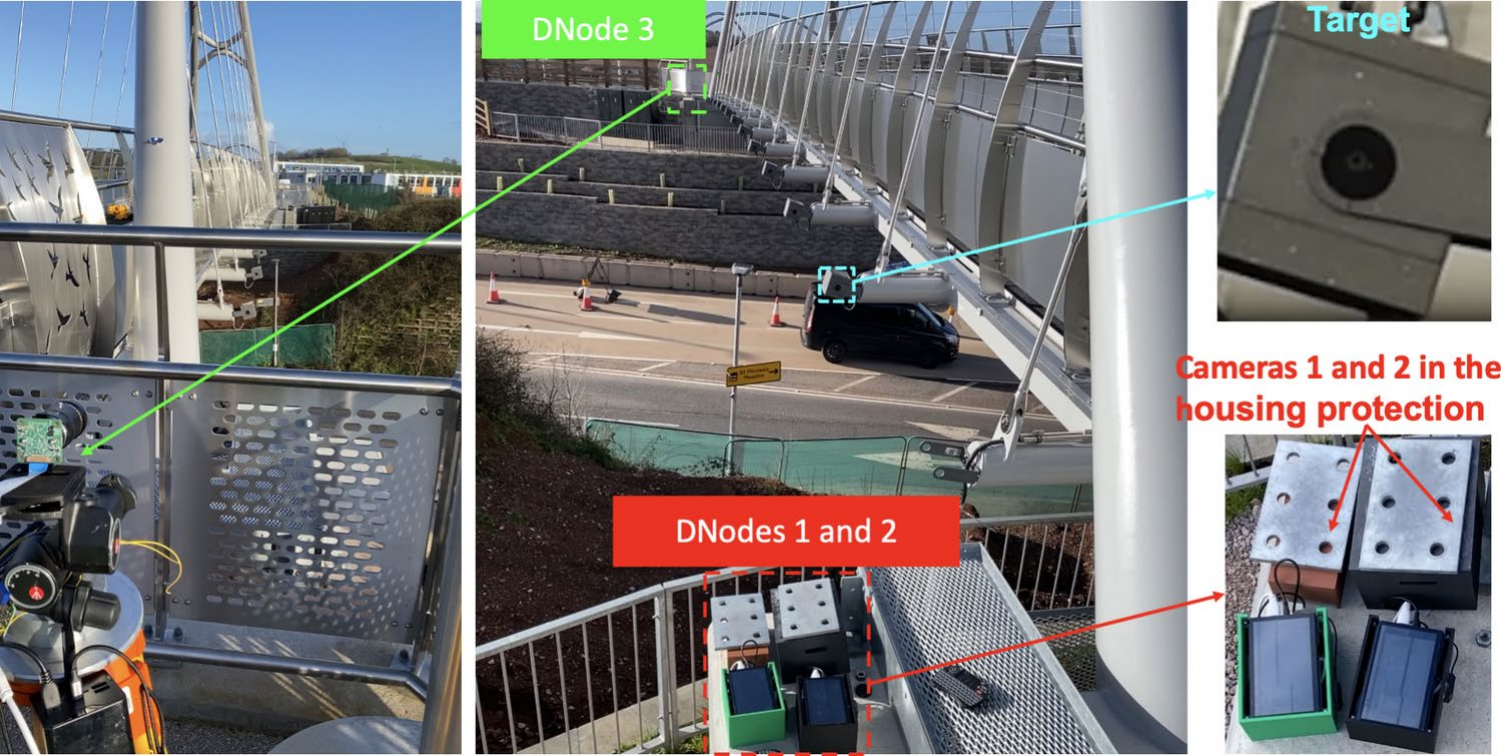
Test setup of the modal testing on an arch footbridge

To capture the vibrations of the entire bridge deck, 12 test points were set at the joints between the bridge deck and the hangers, excluding the two hangers near each end. Three DNodes were used to measure the displacement at these test points, with each node responsible for four points, as illustrated in Fig. 14b. DNodes 1 and 2 were placed on the ground at one end of the bridge, while DNode 3 was mounted on a surveying tripod at the other end. Housing protections were used to shield the cameras of DNodes 1 and 2 from wind-induced movement, as shown in Fig. 15. The system clock of each node was synchronised with satellite clocks via the GNSS antenna and NTP service, ensuring synchronisation among the three nodes regardless of their relative distances.

To avoid disturbing the normal use of the bridge, black circles on the light boxes near the end of each hanger were selected as natural targets for DNode tracking, as shown in the top right of Fig. 15. The height of the light box was 155 mm, which was used to calculate the scaling factor when measuring the corresponding target.

The cameras of DNodes 1 and 3 were equipped with 8–50 mm zoom lenses, while a 75 mm prime lens was used in DNode 2. Since the targets were distributed along the optical axis of the cameras, the aperture of the lenses was adjusted to be minimal, to increase the depth of field. This adjustment ensured that each camera could clearly capture the movement of the corresponding targets. The three nodes were set to record videos at a resolution of 1280 by 720 pixels at 50 Hz. The displacement of each target was then extracted from the corresponding video.

### Test result

**Fig. 16:**
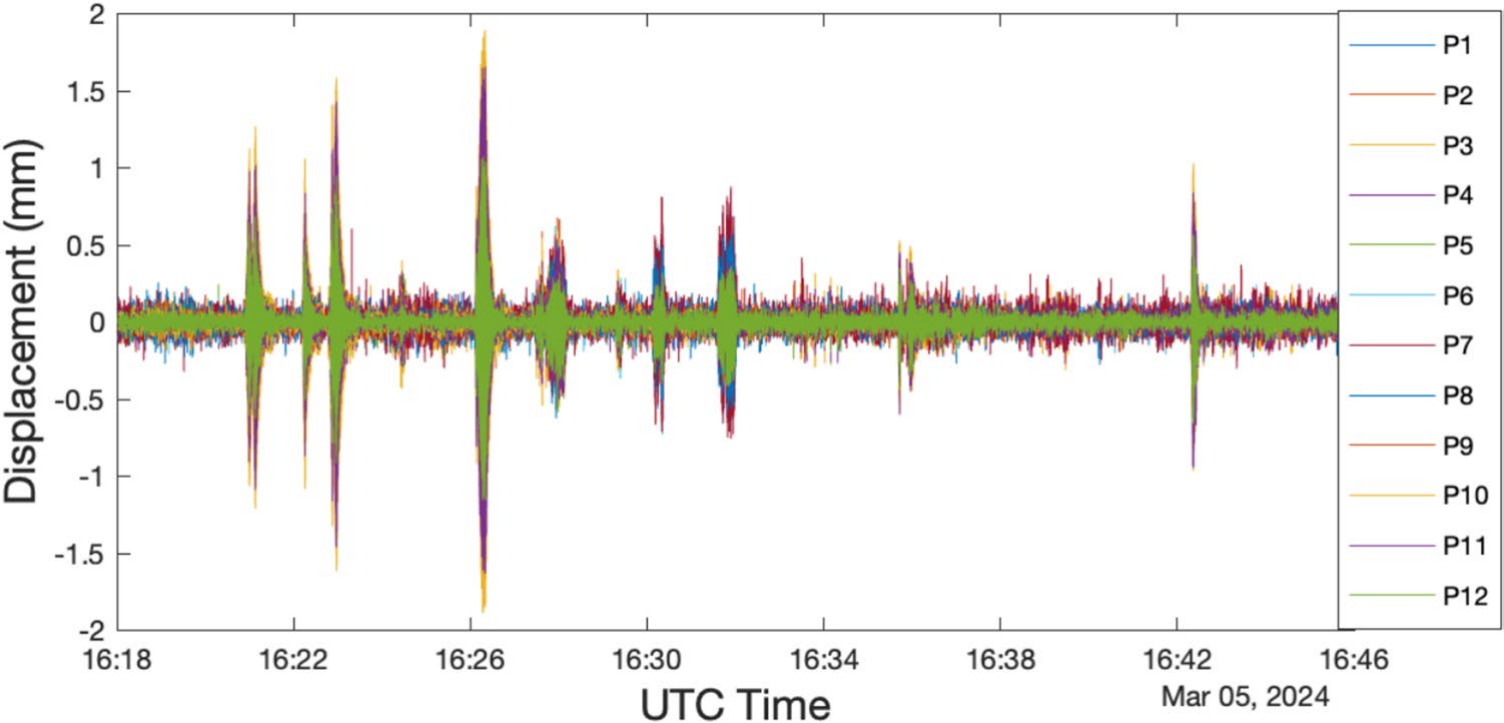
The captured displacement time history of the 12 test points

The test lasted approximately half an hour. During this period, the bridge was excited by human-bouncing movements at the quarter span of the bridge when no other pedestrians were present. These actions were repeated several times, each lasting about 1 min. The displacement signals from 12 points, recorded by three DNodes, are presented in Fig. 16.

After the DNodes completed the recording of the structural vibrations, an ambient modal test using JA accelerometers was conducted on the bridge. Fourteen accelerometers were placed on the bridge deck, distributed across three setups to identify 3D structural modes. The arrangement of the test points for the JA accelerometers is depicted in Fig. 14b, with each setup lasting 10 min.

**Fig. 17:**
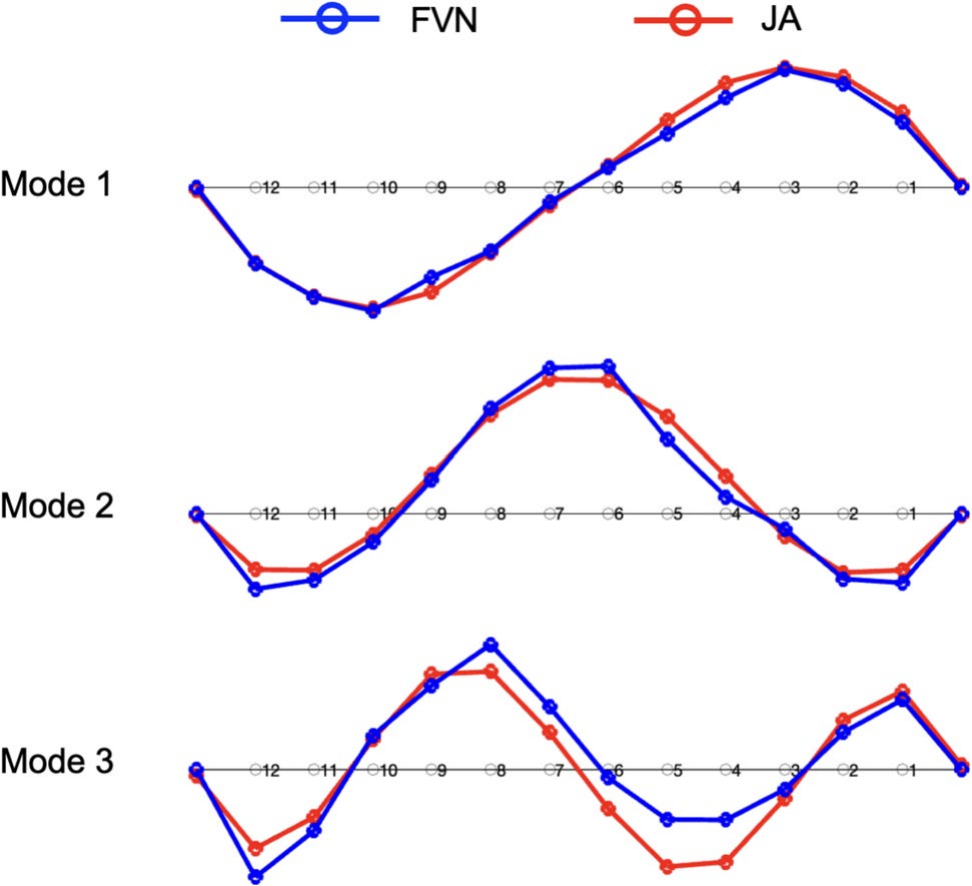
Identified mode shapes based on the FVN and JA data

To determine the structural modal parameters, Bayesian operational modal analysis (BAYOMA) was applied to the data collected from both the FVN and JA systems [37–39]. This analysis identified three vertical bending modes from the FVN data, with the mode shapes illustrated in Fig. 17 and the frequencies listed in Table 6. The first mode, observed at 3.16 Hz, displayed the first-order anti-symmetric vertical bending mode, differing from the symmetric vertical bending mode typically seen in simply supported beam bridges. The subsequent modes were identified as second-order symmetric and anti-symmetric vertical bending modes, with frequencies of 5.36 and 9.23 Hz, respectively.

**Table 6:** The structural frequencies identified based on data obtained by the FVN and JA system

Mode	FVN	JA	Difference	MAC
1	3.16	3.17	−0.32%	0.99
2	5.36	5.32	0.75%	0.98
3	9.23	9.25	−0.22%	0.93

The corresponding mode frequencies derived from JA data are also listed in Table 6. To evaluate the modal assurance criterion (MAC) between the mode shapes identified from the two types of sensor data, the JA mode shape data were adjusted to align with the FVN test points. The frequency differences and MAC values were then calculated and are listed in Table 6. The frequency differences are less than 0.75%, and the MAC values are greater than 0.93, indicating a high correlation between the results obtained from the FVN and JA data. Additionally, the adjusted JA mode shapes are also plotted in Fig. 17.

The MAC value for the third mode is 0.93, the lowest among the identified modes. This shows that the difference in modal amplitude is largest at the highest frequency. The reason is that the displacement data measured at higher frequencies has a relatively low SNR.

Although each setup using accelerometers lasted for 10 min, it took more than half an hour to move and sort these wired sensors for the next setup. The total time for this accelerometer-based modal testing was about 2 h, four times longer than the half-hour single setup required for the FVN modal analysis.

The test results demonstrate that FVN is a viable tool for modal testing on operational arch bridges. Unlike accelerometers, FVN offers a completely non-contact method for capturing the structural vibration modes of bridges, allowing for uninterrupted normal use. Moreover, the modal parameters of the entire structure can be captured in a single setup using fewer FVN nodes and less time compared to accelerometers. However, the vibration data from FVN nodes exhibited a lower signal-to-noise ratio than that from the accelerometers, resulting in fewer identified vibration modes.

## Simultaneous measurement for live loads and structural response on an operational arch footbridge

Input–output monitoring on real-life bridges often involves a variety of sensors. Camera-based systems identify vehicles and pedestrians as input measurements, while accelerometers and displacement transducers measure structural responses. Conventional time synchronisation methods rely on a central controller or data logger to send trigger signals to, or collect data from, each sensor via cables. This approach can be cumbersome in operational bridge applications and poses a significant challenge for input–output SHM. To address these challenges, this section conducted an input–output measurement on the same footbridge discussed in Sect. 5, using a combination of a DNode and LNode.

### Test setup

**Fig. 18:**
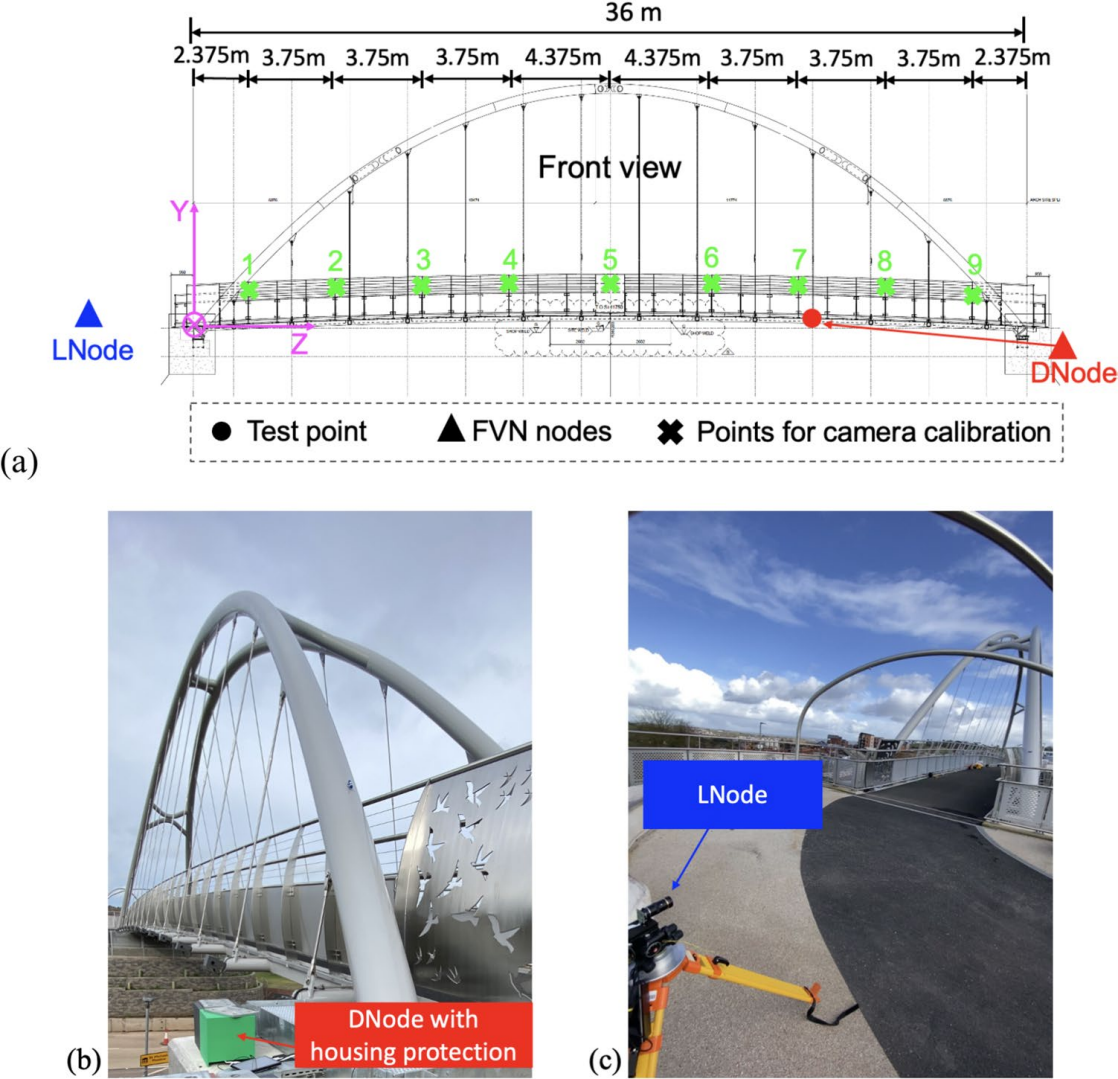
**a** Layout of the test points and FVN nodes, and on-site setup of the two FVN nodes: **b** the DNode and **c** the LNode

The DNode was used to measure live loads, and the LNode to capture structural responses. Figure 18a illustrates the FVN setup in this experiment. The DNode was positioned at one end of the bridge to measure displacement near the quarter span. The LNode was installed on a surveying tripod at the opposite end to identify and position pedestrians on the bridge. The DNode's camera was equipped with a 180 mm fixed focal length lens and encased for wind protection. The camera in the LNode featured an 8–50 mm zoom lens, specifically set to monitor pedestrian movements on the bridge deck, as depicted in Fig. 18b.

Determining the 3D coordinates of pedestrians on bridges with a single camera requires reversing the projection from a 2D pixel plane to a 3D world coordinate system, which necessitates depth information. In this study, pedestrian height was assumed to be known, and depth information was estimated using the pinhole camera model.

**Fig. 19:**
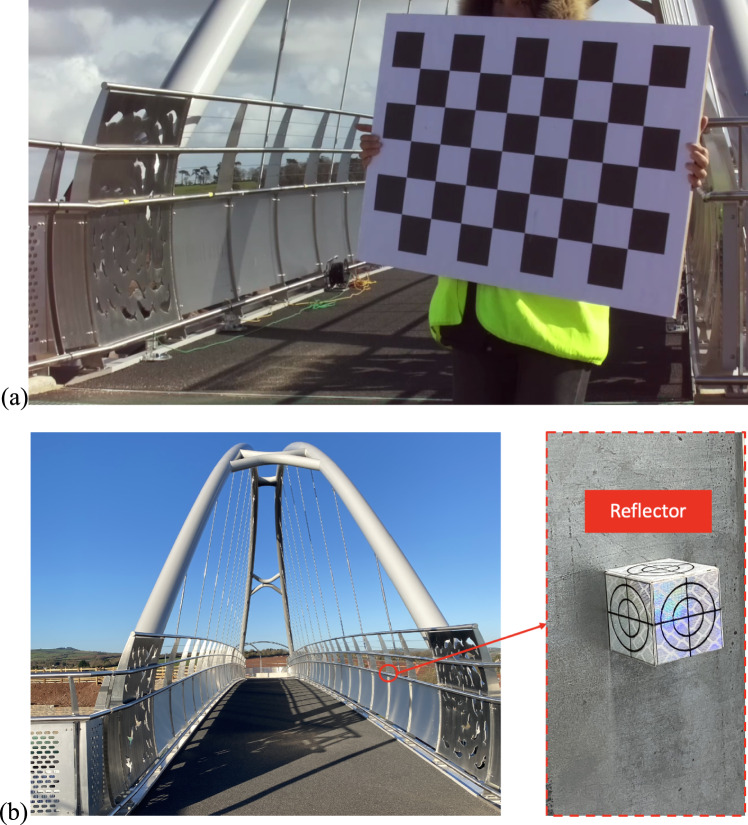
On-site calibration for the LNode: **a** chessboard for camera intrinsic matrix and **b** reflector for camera extrinsic matrix

An on-site camera calibration was performed to obtain the intrinsic matrix and distortion vector of the LNode’s camera using Zhang’s calibration method [33]. A checkerboard with 8*5 inner corners and a grid length of 85 mm served as the calibration tool, as illustrated in Fig. 19a.

To determine the extrinsic matrix of the LNode’s camera, 18 reference points with known coordinates were set on the parapets along both sides of the bridge. The layout of the reference points on one side is illustrated in Fig. 18a. All reference points were positioned 1 m above the bridge deck. Reflectors were placed at the reference points for easier determination of their pixel coordinates in the captured images, as shown in Fig. 19b. During the extrinsic matrix calibration, a torch was used to point at each reflector, creating a white dot on the image. The centre of these white dots was regarded as the pixel coordinate of the reference points. The origin of the 3D world coordinate system was established at one end of the bridge deck, near reference point 1. The Y-axis is defined as upward, the Z-axis points in the direction from reference point 1–9 as shown in Fig. 18a, and the X-axis orientation follows the right-hand rule. The distance between the test point for Node 1 and this origin is 26.75 m.

### Test result

During the test, one person walked from one end of the bridge to the other, while a second person squatted at midspan. The walking person (62.6 kg) was considered to be a live load, and the squatting person (61.8 kg) was treated as a static load at a known location, so tracking the position of the squatting person was unnecessary. Initially, the walking person ran from the end of the bridge towards the midspan, then slowed to a walk near the midpoint, continuing the remainder of the path at a walking pace.

**Fig. 20:**
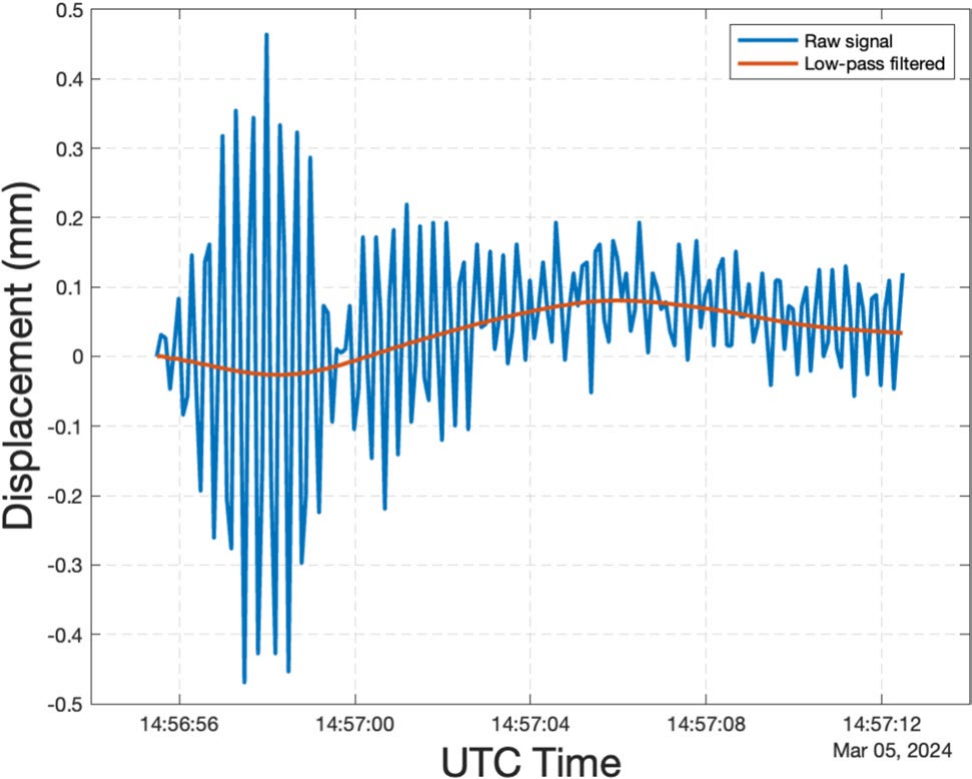
Measured structural displacement response at the test point near the quarter span

The structural displacement response, primarily induced by the moving person, was monitored in real time by the DNode, as shown in Fig. 20. The displacement signal contained both dynamic and static components; the dynamic component arose from the periodic impact of human walking, while the static component resulted from the weight of the two people. To analyse the bridge deformation at the test point caused by the moving person's varying positions, a low-pass filter with a cutoff frequency of 0.1 Hz was applied to the raw data. This processing yielded a quasi-static displacement profile, shown in Fig. 20, where the structural deformation under the combined weight of both individuals was minimal, peaking at less than 0.1 mm.

**Fig. 21:**
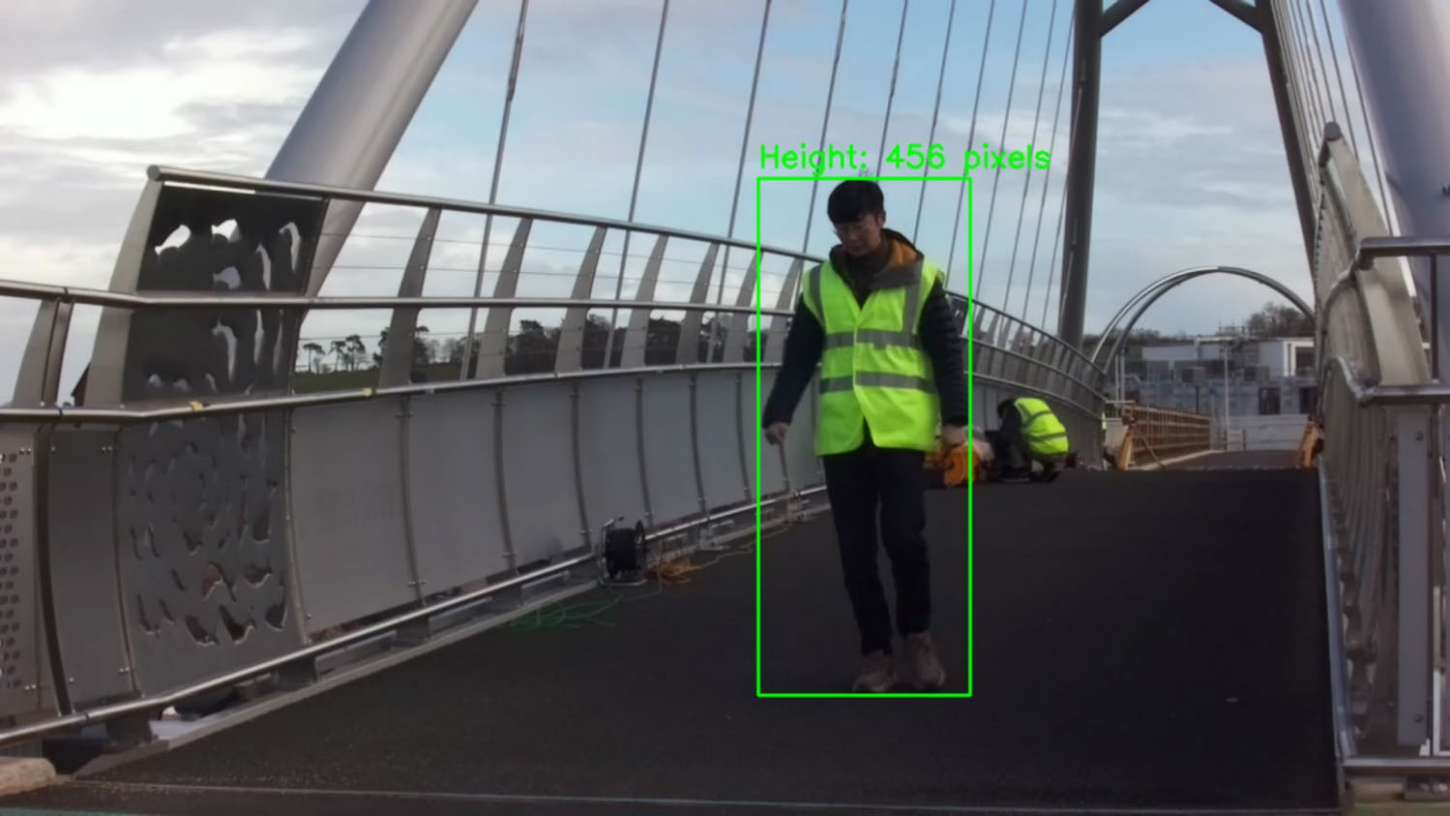
Pedestrian identification using YOLO v8 with mark of the height of the bounding box

To estimate the moving person's depth information, YOLO v8 was employed to identify this individual and mark him with a bounding box, as illustrated in Fig. 21. The person's height, including shoes, was recorded at 177 cm. Using this height, along with the bounding box dimensions and the lens focal length, the moving person's depth information was estimated. Subsequently, the 3D coordinates of this moving individual on the bridge were computed using both camera intrinsic and extrinsic matrices, supplemented by the depth data.

**Fig. 22:**
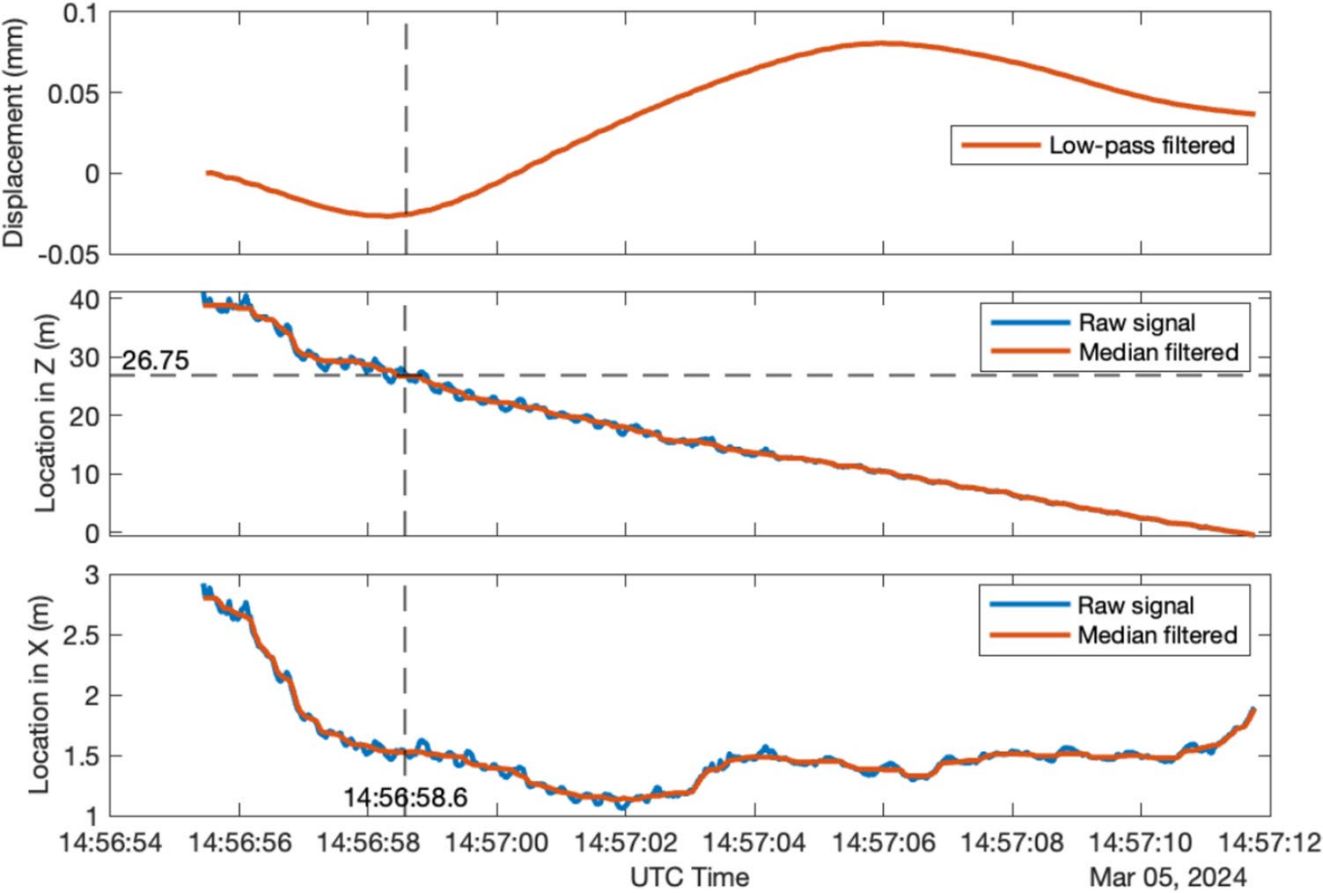
3D locations of the moving person during walking and the corresponding structural quasi-static displacement

Figure 22 illustrates the locations of the moving person along the X-axis and Z-axis over time as he crossed the bridge. During pedestrian identification using YOLO v8, the entire body of the pedestrian is enclosed within a bounding box, causing the height of the bounding box to increase when the pedestrian stands on tiptoe. Notably, the height of the bounding box varied with each step as the person walked, introducing errors in calculating the 3D coordinates. To reduce these errors, a median filter was applied to the initial results, as depicted in Fig. 22.

The corresponding structural quasi-static displacement filtered from the measured displacement signal is also displayed in the first subplot of Fig. 22. The shape of the quasi-static displacement is similar, with the theoretical displacement influence line of arch bridges at the quarter span [40]. At 14:56:58.6, as the person reached the test point with a Z-axis value of 26.75 m, the observed quasi-static displacement was −0.026 mm, approaching the maximum downward displacement.

These results showcase the capability of FVN to measure small structural displacements and locate live loads, demonstrating the potential of FVN for input–output measurements on operational footbridges. However, calculating the 3D coordinates of pedestrians using a single camera presents significant challenges. The method used in this study for estimating 3D coordinates lacks the precision required for high-accuracy location measurements of pedestrians. The primary objective of this test was to demonstrate the potential of our FVN system for input–output measurements, rather than to precisely position pedestrians on the bridge. Employing multi-view human pose estimation with multiple cameras could address this issue and enhance accuracy.

## Conclusions

This study has developed a wireless vision sensor network, the flexible vision network (FVN), for SHM. It includes synchronisation and measurement accuracy validation tests, as well as various applications on full-scale structures. The main conclusions are as follows:

1. The error of the GNSS-based synchronisation method was evaluated by comparing the system clocks of two Raspberry Pi units synchronised with satellite clocks via a GNSS module over a 24 h test period. The synchronisation errors were predominantly kept under 100 µs, achieving a standard deviation ($\sigma$) of 17 µs.
2. The time difference between two displacement signals of a shared target, measured by two DNodes with rolling shutter cameras (Raspberry Pi High Quality cameras), was less than 3.05 ms. With global shutter cameras (Raspberry Pi Global Shutter Cameras), the difference was controlled to under 0.34 ms. This result suggests an existence of major timestamping uncertainty in the cameras.
3. DNodes can capture accurate structural displacement with a precision greater than 1/37 pixels when using rolling shutter cameras and 1/65 pixels when using global shutter cameras.
4. It is feasible to use multiple DNodes for operational modal analysis of full-scale structures with small vibrational displacements.
5. FVN can be used in input–output measurements, with LNodes estimating the location of moving loads on the structures and DNodes measuring the resulting structural displacements.

The test described in Sect. 6 used only one LNode to estimate the position of a pedestrian on a footbridge, rather than estimating dynamic walking forces. Future work will employ multiple LNodes to accurately construct a 3D pedestrian body model during walking. The pedestrian’s gait forces can then be estimated from the accelerations of the model’s feet, facilitating comprehensive input–output structural analysis.

## Data Availability

Data will be made available on request.
